# Serum B cell–activating factor (BAFF) level in connective tissue disease associated interstitial lung disease

**DOI:** 10.1186/s12890-015-0105-0

**Published:** 2015-09-30

**Authors:** Tsutomu Hamada, Takuya Samukawa, Tomohiro Kumamoto, Kazuhito Hatanaka, Go Tsukuya, Masuki Yamamoto, Kentaro Machida, Masaki Watanabe, Keiko Mizuno, Ikkou Higashimoto, Yoshikazu Inoue, Hiromasa Inoue

**Affiliations:** Department of Pulmonary Medicine, Graduate School of Medical and Dental Sciences, Kagoshima University, 8-35-1 Sakuragaoka, Kagoshima, 890-8520 Japan; Department of Molecular and Cellular Pathology, Graduate School of Medical and Dental Sciences, Kagoshima University, Kagoshima, Japan; Clinical Research Center, National Hospital Organization Kinki-Chuo Chest Medical Center, Osaka, Japan

**Keywords:** BAFF, Biomarker, CTD-ILD, CFIP

## Abstract

**Background:**

Interstitial lung diseases (ILDs) are common in patients with connective tissue diseases (CTDs). Although the diagnosis of an underlying CTD in ILD (CTD-ILD) affects both prognosis and treatment, it is sometimes difficult to distinguish CTD-ILD from chronic fibrosing interstitial pneumonia (CFIP). B cell–activating factor belonging to the tumour necrosis factor family (BAFF) plays a crucial role in B cell development, survival, and antibody production.

**Methods:**

We examined serum levels of BAFF, surfactant protein D (SP-D), and Krebs von den Lungen-6 (KL-6) in 33 patients with CTD-ILD, 16 patients with undifferentiated CTD-ILD, 19 patients with CFIP, and 26 healthy volunteers. And we analysed the relationship between serum BAFF levels and pulmonary function, as well as the expression of BAFF in the lung tissue of patients with CTD-ILD.

**Results:**

Serum levels of BAFF were significantly higher in CTD-ILD patients compared to healthy subjects and CFIP patients. However, there were no significant differences in serum levels of SP-D and KL-6. Furthermore, serum BAFF levels in CTD-ILD patients were inversely correlated with pulmonary function. BAFF was strongly expressed in the lungs of CTD-ILD patients, but weakly in normal lungs.

**Discussion:**

This is the first study to demonstrate that serum BAFF levels were significantly higher in CTD-ILD patients compared to healthy subjects and CFIP patients. Furthermore, serum BAFF levels were correlated with pulmonary function. We consider that serum BAFF levels in patients with CTD-ILD reflect the presence of ILDs disease activity and severity.

**Conclusion:**

These finding suggest that BAFF may be a useful marker for distinguishing CTD-ILD from CFIP.

## Background

Connective tissue diseases (CTDs) are inflammatory, immune-mediated disorders that can cause a large variety of pulmonary complications, including bronchiolitis, pleuritis, and pulmonary hypertension. Interstitial lung diseases (ILDs) are a common form of pulmonary involvement associated with CTDs that is characterised by various patterns of inflammation and fibrosis [1]. Interstitial lung involvements of CTD are usually referred to as interstitial pneumonia, which is further subdivided into several histopathological or radiological entities, including usual interstitial pneumonia (UIP), nonspecific interstitial pneumonia (NSIP), and organizing pneumonia (OP).

The prognosis of patients with CTD-ILD is generally more favourable than those with idiopathic interstitial pneumonias (IIPs) [1–3]. Some patients with progressive CTD-ILD may benefit from corticosteroids, immunosuppressive therapy, or both [4, 5]. Since a diagnosis of underlying CTDs affects prognosis and treatment, it is crucial to evaluate for underlying CTDs in patients with ILDs. Although clinical evaluation and measurement of a variety of autoantibody titres are recommended in the evaluation for underlying CTDs in patients with ILDs, it is sometimes difficult to distinguish CTD-ILD from chronic fibrosing interstitial pneumonia (CFIP), which including idiopathic pulmonary fibrosis (IPF) and idiopathic NSIP [6]. Recent reports have described a population of ILDs patients with clinical features of CTDs with or without autoantibodies who do not meet criteria for a defined CTDs. This population has been referred to by several names, including undifferentiated CTD-ILD (UCTD-ILD), lung dominant CTD, and autoimmune-featured ILD [1, 7, 8].

Several studies have suggested that serum biomarkers, such as surfactant protein A (SP-A), surfactant protein D (SP-D), and Krebs von den Lungen-6 (KL-6), are useful not only for the diagnosis of ILDs but also for evaluation of disease activity [6]. However, there are no studies on whether these biomarkers could be useful for distinguishing CTD-ILD from CFIP. Markers with higher specificity may serve as integral components of the evaluation for CTD-ILD.

B cell–activating factor belonging to the tumour necrosis factor family (BAFF) is responsible for B-cell survival and maturation [9]. BAFF is expressed by monocytes, macrophages, dendritic cells, and T cells. *BAFF* encodes a putative 285-amino acid type II transmembrane protein that is biologically active as a cell surface protein or in a soluble form [10]. BAFF is believed to play a role in autoantibody production and is considered a promoting factor in the pathogenesis of several autoimmune diseases [11]. Elevated serum levels of BAFF have been reported in patients with systemic lupus erythematosus [12], rheumatoid arthritis [13], Sjögren's syndrome [14], systemic sclerosis [15], and dermatomyositis [16]. Serum BAFF levels are associated with disease activity for these clinical entities. Furthermore, it has been shown the myositis and mixed CTD (MCTD) patients with ILDs have significantly higher BAFF levels than those without ILDs [16, 17]. A recent study showed plasma BAFF concentrations are significantly higher in IPF patients than in either COPD patients or control subjects [18]. However, serum BAFF levels has never been evaluated in patients with CFIP and compared with CTD-ILD.

Therefore, we measured serum BAFF levels and evaluated the clinical features of patients with CFIP, UCTD-ILD, and CTD-ILD, in order to evaluate the utility of BAFF in detecting CTDs in patients presenting with ILDs.

## Methods

### Study subjects

Characteristics of ILDs patients enrolled in this study are shown in Table 1. Sixty-eight patients (35 males and 33 females aged 65.1 ± 9.2 y) were diagnosed with ILDs, including 19 with CFIP (11 with IPF, 8 with idiopathic NSIP), 16 with UCTD-ILD, and 33 with CTD-ILD. All consecutive ILDs patients seen at Kagoshima University Hospital (Kagoshima, Japan) from 2008 to 2012 were included in this study. The diagnosis of CFIP was based on clinical, radiographic, and pulmonary physiological features, according to the American Thoracic Society/European Respiratory Society consensus classification [6, 19]. Histological classification of ILDs was based on pathologic findings in surgical lung biopsy specimens. Underlying CTDs consisted of rheumatoid arthritis (*n* = 5), dermatomyositis (*n* = 7), systemic sclerosis (*n* = 16), Sjögren's syndrome (*n* = 1), systemic lupus erythematosus (*n* = 1) and MCTD (*n* = 3). We used American College of Rheumatology (ACR) criteria for the diagnosis of rheumatoid arthritis, systemic sclerosis, Sjögren's syndrome, and systemic lupus erythematosus. For the diagnosis of dermatomyositis and MCTD, we used the other criteria [20, 21]. Patients were considered to have UCTD-ILD if signs or symptoms or laboratory findings in their medical record met Kinder’s criteria for UCTD (*n* = 14) [7]. Patients with lung cancer, environmental exposures and other known causes of ILDs were excluded. All serum samples were collected at the time of diagnosis, prior to the initiation of systemic steroid or immunosuppressive therapy, and stored at −80 °C until this investigation. Twenty-six healthy volunteers with normal chest radiographs not taking any medications were enrolled in our study as a control group. The Human Ethics Review Committee of Kagoshima University School of Medicine approved the study protocol and all participants provided written informed consent prior to enrolment in the study.

**Table 1:** Characteristics of patients with interstitial lung disease associated with connective tissue disease (CTD-ILD), undifferentiated CTD-ILD (UCTD-ILD), chronic fibrosing interstitial lung disease (CFIP), and control subjects

	Control	CFIP	UCTD-ILD	CTD-ILD	*p*-value
Subjects, *n*	26	19	16	33	
Age, years	64 (59–81)	67 (54–80)	70 (53–79)	63 (39–83)	0.0265
Females, *n* (%)	16 (61)	3 (15)	8 (50)	22 (66)	0.0032
Smoking status					
Former smoker, *n* (%)	7 (26)	12 (63)	8 (50)	11 (33)	0.0617
Current smoker, *n* (%)	1 (3)	2 (10)	1 (6)	0 (0)	0.3250
Pack-years	0 (0–40)	30 (0–150)	5 (0–70)	0 (0–100)	0.0035
*P*_a, O2_ mmHg		83.6 (62.4–111.7)	72.5 (58.8–86.6)	79.2 (42.3–105.2)	0.1413
KL-6, U · mL^−1^	261 (140–491)	1581 (304–8298)	1045 (363–4560)	1117 (289–3943)	<0.0001
SP-D, ng · mL^−1^	32.3 (8.6–126.0)	292.5 (32.3–1070)	206.0 (88.6–405)	175.0 (31.2–994.0)	<0.0001
BAFF, ng · mL^−1^	0.6 (0.5–0.9)	1.0 (0.3–1.9)	1.8 (1.0–2.3)	2.0 (0.6–16.7)	<0.0001
VC, L		1.9 (0.9–3.1)	2.1 (0.9–3.1)	2.0 (1.0–3.5)	0.9213
VC, % pred		59.8 (35.4–128.2)	69.2 (37.9–97.4)	65.4 (41.3–104.1)	0.3390
DL,_CO,_ mL · min^−1^ · mmHg^−1^		9.3 (3.4–15.4)	8.9 (4.5–16.9)	10.8 (4.6–16.9)	0.6097
DL,_CO,_ %pred		63.2 (43.5–125.4)	60.4 (26.9–74.4)	64.7 (29.0–109.4)	0.2751

Data are presented as counts (n) or medians and ranges (minimum-to-maximum values), unless otherwise stated. Differences in each variable between the various groups were analysed using the Kruskal-Wallis test. *P*_a,O2_: oxygen tension in arterial blood; SP-D: surfactant protein D; KL-6: Krebs von den Lungen-6; BAFF: B cell–activating factor belonging to the tumour necrosis factor family; VC: vital capacity; % pred: % predicted; DL,_CO_: diffusing capacity of the lung for carbon monoxide

### Pulmonary function test

Pulmonary function test, including vital capacity (VC), forced vital capacity (FVC), and forced expiratory volume in 1 s was performed using an electrical spirometer. Predicted FVC values (FVC, % predicted) were calculated. Diffusing capacity for carbon monoxide (DL_CO_) was measured using the single-breath technique or the re-breathing technique, which was then adjusted to single-breath values.

### Enzyme-linked immunosorbent assay (ELISA)

Serum BAFF was quantified using a sandwich ELISA in a commercially available kit (Human BAFF ELISA Kit, R&D Systems, Minneapolis, MN, USA). The lower limit of detection was 2.43 pg•mL^−1^. Serum samples with BAFF levels exceeding the maximum value of the standard curve for the kit were diluted and re-assayed. Serum KL-6, SP-D, and SP-A were measured using commercially available ELISA kits (Eitest KL-6 kit, Sanko Junyaku, Tokyo, Japan; SP-D kit, YAMASA EIA, Yamasa, Japan; and SP-A test Kokusai-F kit, International Reagents Corporation, Kobe, Japan) [22–24].

### Immunohistochemical staining of BAFF in lung tissue

We investigated BAFF expression in surgical biopsy specimens obtained from four never-smoker patients each with CFIP, UCTD, and CTD-ILD. Normal lung tissue was obtained from a non-smoker who underwent extirpation of a postoperative thoracic empyema at Kagoshima University Hospital. Lung tissue was fixed in 10 % neutral buffered formalin for 24 to 72 h and embedded in paraffin. After blocking the endogenous peroxidase activity, deparaffinised sections (3 μm thick) were pretreated in 1 mM EDTA buffer (pH 8) and microwaved (600 W) for 8 min. After cooling for 30 min, the sections were incubated with goat polyclonal anti-human BAFF/BLyS/TNFSF13B antibody (R&D Systems) diluted to 1:40 for 30 min using the DAKO LSAB2 system (DAKO Cytomation, Glostrup, Denmark). To visualise immunoreactivity, DAB+ (DAKO Cytomation) was used. Non-immune serum was used instead of the primary antibody as a negative control.

### Statistical analysis

Since the data were not normally distributed, non-parametric tests were used for all comparisons. Differences in each variable between the various groups were first analysed using the Kruskal-Wallis test, followed by the Bonferroni test. Fisher's exact test was used to determine group differences. We considered p values of <0.05 as significant. Statistical analysis was performed using Graph Pad Prism 6 (Graph Pad, San Diego, CA, USA).

## Results

### Characteristics of the study participants

Table 1 shows the characteristics of the participants in the control group and patients with CFIP, UCTD-ILD, or CTD-ILD. Data are shown as medians and ranges.

### Serum BAFF levels in patients with CTD-ILD compared to patients with CFIP or UCTD and control subjects

**Fig. 1:**
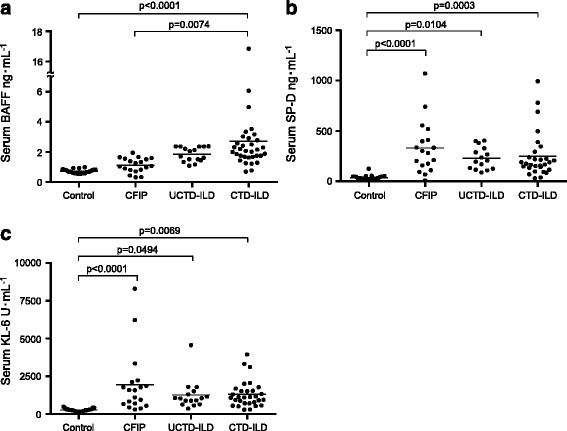
Distribution of serum **a** BAFF, (**b**) SP-D, and (**c**) KL-6 levels in patients with CTD-ILD (*n* = 33), undifferentiated CTD-ILD (*n* = 16), CFIP (*n* = 19), and healthy control subjects (*n* = 26). Differences in each variable across groups were first analyzed using the Kruskal-Wallis test, followed by the Bonferroni test. BAFF: B cell–activating factor belonging to the tumour necrosis factor family; KL-6: Krebs von den Lungen-6; SP-D: surfactant protein D; CTD-ILD: interstitial lung disease associated with connective tissue disease; CFIP: chronic fibrosing interstitial lung disease

Serum levels of BAFF, SP-D and KL-6 are shown in Fig. 1. Serum KL-6 levels were significantly higher in patients with CFIP (*n* = 19; 1581 (304–8298) U•mL^−1^, *p* < 0.0001), UCTD-ILD (*n* = 16; 1045 (363–4560) U•mL^−1^, *p* = 0.0494), and CTD-ILD (*n* = 33; 1117 (289–3943) U•mL^−1^, *p* = 0.0069) than in control subjects (*n* = 26; 261 (140–491) U•mL^−1^). Similarly, patients with CFIP (*n* = 19; 292.5 (32.3-1070) ng•mL^−1^, *p* < 0.0001), UCTD-ILD (*n* = 16; 206.0 (88.6-405) ng•mL^−1^, *p* = 0.0104), and CTD-ILD (*n* = 33; 175.0 (31.2-994.0) ng•mL^−1^, *p* = 0.0003) had significantly higher SP-D levels than control subjects (*n* = 26; 32.3 (8.6-126.0) ng•mL^−1^). However, there were no differences among patients with CTD-ILD, UCTD-ILD, and CFIP. Conversely, serum BAFF levels were significantly higher in patients with CTD-ILD (*n* = 33; 2.0 (0.6-16.7) ng•mL^−1^) than in patients with CFIP (*n* = 19; 1.0 (0.3-1.9) ng•mL^−1^, *p* = 0.0074) or healthy controls (*n* = 26; 0.6 (0.5-0.9) ng•mL^−1^, *p* < 0.0001).

### Relationship between BAFF levels and clinical parameters in patients with CTD-ILD

**Fig. 2:**
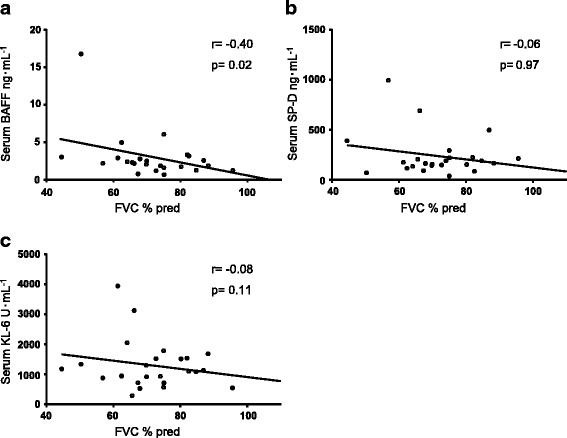
Correlation between serum BAFF, SP-D, and KL-6 levels and pulmonary function testing parameters in patients with CTD-ILD. The correlation between serum BAFF (**a**), SP-D (**b**), and KL-6 (**c**) and FVC % predicted in CTD-ILD patients is shown. BAFF: B cell–activating factor belonging to the tumour necrosis factor family; SP-D: surfactant protein D; KL-6: Krebs von den Lungen-6; FVC: forced vital capacity; CTD-ILD: interstitial lung disease associated with connective tissue disease

We analysed the relationship between pulmonary function testing parameters and markers of interstitial lung disease (Fig. 2). Serum BAFF levels are inversely correlated with VC, % predicted (r = −0.40, *p* < 0.05) in patients with CTD-ILD. However, there were no significant correlations between pulmonary function testing parameters and serum SP-D and KL-6 levels. Sex, smoking status and serum SP-D and KL-6 were not associated with serum BAFF levels. There were no significant correlation between serum BAFF levels and other biomarkers (data were not shown).

### ROC analysis comparing serum BAFF, KL-6, and SP-D levels between patients with CTD-ILD and CFIP

Compared to control subjects or CFIP patients, serum BAFF levels were significantly higher in CTD-ILD patients. We evaluated the sensitivity and specificity of various serum biomarkers for distinguishing patients with CTD-ILD from control subjects or CFIP patients using ROC curve analysis (Table 2). First, the areas under the ROC curve (AUC) for patients with CTD-ILD versus controls were 0.972 for serum BAFF, 0.960 for SP-D, and 0.983 for KL-6. ROC curve analysis showed that serum BAFF, SP-D and KL-6 levels had good specificity (BAFF, 95.2 %; SP-D 95.0 %; KL-6, 100 %) and good sensitivity (BAFF, 91.1 %; SP-D, 94.1 %; KL-6, 94.1 %). We then evaluated the ROC curves for these markers in patients with CTD-ILD and CFIP. AUC was highest for BAFF: serum BAFF, 0.832; SP-D, 0.356; and KL-6, 0.458. The value of BAFF closest to 100 % sensitivity and 100 % specificity selected as the cut-off value was 1.7 μg•mL^−1^ (sensitivity, 67 %; specificity, 94 %). Use of this serum BAFF cut-off level result in a low false-positive rate for the diagnosis of CTD-ILD among CFIP patients (5 %, 1/19), however, false-negative in 11 of 33 patients with CTD-ILD. However, no differences were found in the presence of autoantibodies from individuals with below or above the cut-off level. Overall, using serum BAFF in the diagnosis of CTD-ILD yielded the highest diagnostic accuracy and the greatest sensitivity, specificity, and likelihood ratio among these markers. These results demonstrate that patients with CTD-ILD can be distinguished from controls or CFIP patients using measurements of serum BAFF levels.

**Table 2:** ROC analysis comparing serum BAFF, KL-6, and SP-D levels between patients with CTD-ILD and CFIP

	AUC	Cut-off value	Sensitivity, % (95 % CI)	Specificity, % (95 % CI)	Likelihood ratio
BAFF	0.823	1.7 ng · mL^−1^	67.6 (49.4 to 82.6)	94.7 (73.9 to 99.8)	12.85
KL-6	0.566	1562 U · mL^−1^	79.4 (62.1 to 91.3)	52.6 (28.8 to 75.5)	1.67
SP-D	0.643	158 ng · mL^−1^	47.1 (29.7 to 64.8)	77.7 (52.3 to 93.5)	2.11

BAFF: B cell–activating factor belonging to the tumour necrosis factor family; SP-D: surfactant protein D; KL-6: Krebs von den Lungen-6; CTD-ILD: interstitial lung disease associated with connective tissue disease; CFIP: chronic fibrosing interstitial lung disease; ROC: receiver operating characteristic; AUC: area under the ROC curve; CI: Confidence interval

### Immunohistochemical staining for BAFF

**Fig. 3:**
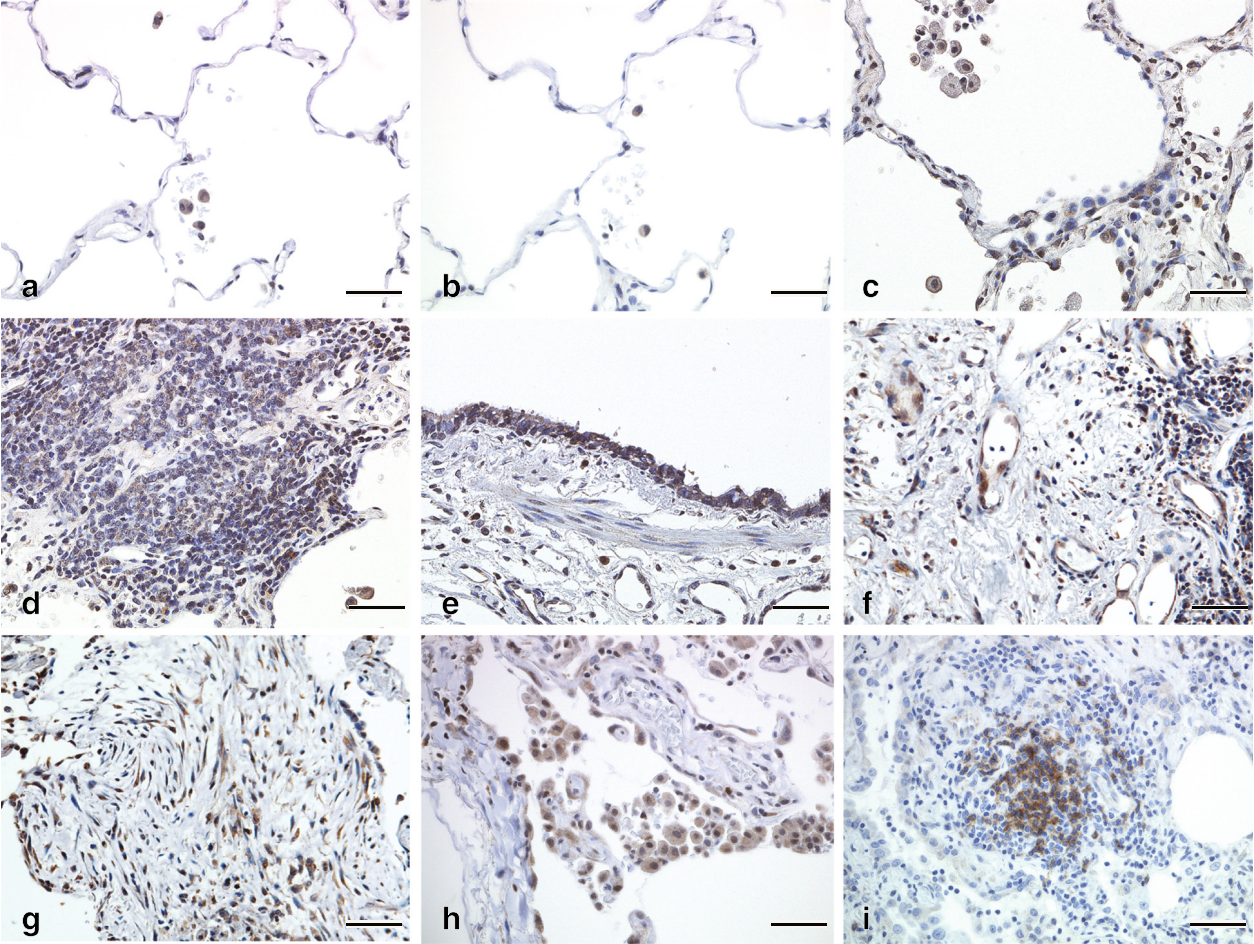
Representative immunohistochemical findings for BAFF in lung sections. Tissue was obtained from a, b a 23-year-old female non-smoker as a control, (**c**–**g**) a 56-year-old female non-smoking patient with CTD-ILD, and (**h**, **i**) a 63-year-old male non-smoking patients with CFIP. Weakly positive alveolar macrophages were observed in the **a**) BAFF **b**) goat IgG control. Strong cytoplasmic positivity of BAFF was observed in **c**), **h**) peripheral airways macrophages and cells in the alveolar walls, **d**), **i**) lymphoid follicles, **e**) peripheral airways, **f**) vascular endothelial cell and **g**) fibroblasts. Scale bars = 50 μm. BAFF: B cell–activating factor belonging to the tumour necrosis factor family; CTD-ILD: interstitial lung disease associated with connective tissue disease; CFIP: chronic fibrosing interstitial lung disease

Figure 3 shows representative BAFF immunohistochemical staining of lung specimens obtained from patients with CTD-ILD and normal areas of lungs surgically removed to treat thoracic empyema. In lung specimens from patients with CTD-ILD, BAFF was clearly overexpressed, mainly in alveolar macrophages in the airspace and infiltrating lymphoid cells, including those arranged in follicular patterns. BAFF positivity was also detected in the cytoplasm of the epithelial cells of the peripheral airways, endothelial cells in blood vessels and fibroblasts (Fig. 3b-g). BAFF was expressed weakly in normal lung tissues (Fig. 3a), which considered as non-specific staining compared with polyclonal goat IgG control (Fig. 3b). There were no clear differences in the expression of BAFF in lung specimen from patients with CTD-ILD versus CFIP (Fig. 3h,-i).

## Discussion

We demonstrated that serum BAFF levels in patients with CTD-ILD were significantly higher than in patients with CFIP and healthy controls. Immunohistochemically, lung sections from patients with CTD-ILD are strongly positive for BAFF in cells of the alveolar walls, peripheral airways, and lymphoid follicles. Moreover, there was a significant correlation between serum BAFF levels and disease severity in patients with CTD-ILD.

Several biomarkers for the diagnosis of ILDs have been found. SP-D and KL-6 levels have been shown to be higher in all of major types of CFIP. One study found serum SP-A and SP-D levels were significantly higher in IPF compared to NSIP/cryptogenic OP or CTD-ILD [25, 26]. However, the specificity of these biomarkers has not been evaluated for CTD-ILD, UCTD, lung dominant CTD, and autoimmune-featured ILD. In the present study, serum SP-D and KL-6 levels in patients with CTD-ILD were similar to those in CFIP patients, and there was no relationship observed between serum SP-D or KL-6 levels and disease activity. Conversely, BAFF was specific for CTD-ILD, and we expect it could be a useful clinical biomarker for distinguishing CTD-ILD from CFIP.

For patients with ILDs, current guidelines recommend an evaluation for underlying CTD. This evaluation will yield a subset of ILD patients with symptoms and autoantibodies suggestive of an autoimmune condition who do not fulfil ACR criteria for CTDs. These patients, in whom the lung is the only or most clinically important manifestation of occult CTDs, are suspected of having a systemic autoimmune disease due to the presence of circulating autoantibodies, specific histopathological features on surgical lung biopsy samples, or subtle extrathoracic manifestations. They could be possibly be classified as having a subtype of CTD-ILD rather than CFIP [27]. The proposed terminology for such patients includes undifferentiated CTD-ILD [7], lung-dominant CTD [27], and autoimmune-featured ILD [8]. Strategies for identifying and diagnosing these patients are controversial and confusing. One reason is that multiple specific autoantibodies are needed for diagnosing UCTD since it includes multiple autoimmune diseases. Our findings show that patients who met Kinder’s criteria for UCTD had significantly higher serum BAFF levels than patients with CFIP; however, the levels of SP-D and KL-6 in patients with UCTD and CFIP were similar. We presume that serum BAFF levels in patients with UCTD-ILD might be higher than in patients with CFIP, and these patients would be classified as having CTD-ILD or a possible subtype of CTD-ILD based on high serum BAFF levels. Previous study showed plasma BAFF concentrations are significantly higher in IPF patients than in control subjects [18]. However, our present study showed serum BAFF levels are significantly higher in CTD-ILD patients than in CFIP patients (which include IPF and idiopathic NSIP). In addition, high serum BAFF levels in ILDs patients may guide initial therapy and prognosis, since treatment improves survival in patients with CTD-ILD compared to those with idiopathic ILDs [11]. NSIP is the most common histological finding in patients with CTD-ILD who meet established ACR criteria, and CTD-ILD is often accompanied by fibrotic interstitial pneumonia that resembles IIPs [11]. Additionally, it has been shown that most patients diagnosed with idiopathic NSIP meet the UCTD of Kinder et al. [7]. The distinction between idiopathic NSIP and IPF has important prognostic and treatment implication [28]. However, histologically distinguishing NSIP with CTD-ILD from NSIP with CFIP is difficult. There are reports that 10 % and 52 % of patients who were considered to have idiopathic NSIP developed CTDs during follow-up [29, 30]. In addition to various specific autoantibody titres, serum BAFF levels might be helpful for distinguishing CTD-ILD and UCTD from CFIP.

This is the first study to demonstrate that patients with CTD-ILD have significantly higher serum BAFF levels than patients with CFIP and healthy controls, as well as significant correlations between serum BAFF levels and parameters indicating disease severity such as VC, % predicted and DL_CO_, % predicted. BAFF immunoreactivity was detected in alveolar macrophages and infiltrating lymphoid cells in patients with COPD in a prior study [31]. In this study, we found overexpression of BAFF in patients with CTD-ILD, mainly in alveolar macrophages in the air space, parenchymal lymphoid follicles, fibroblasts and alveolar walls. Previous studies have found increased serum levels of BAFF in a subset of patients with idiopathic inflammatory myopathies or MCTD [16, 17] compared to healthy individuals, and that patients with ILDs had higher BAFF levels than patients without ILDs. Taken together, we consider that serum BAFF levels in patients with CTD-ILD and UCTD reflect the presence of ILDs activity and severity.

Belimumab is a purely human monoclonal antibody that targets BAFF. It has been used for the first targeted biological treatment approved specifically for systemic lupus erythematosus [32]. Our findings suggest that BAFF might be a new potential target for therapy in patients with CTD-ILD and UCTD-ILD.

Our study has several limitations. First, our study was retrospective in nature. Second, our sample size was limited and our results represent the experience of only a single centre. Therefore, a multicentre study with a larger cohort will be required to confirm our results. We will also need to clarify the relationship between these biomarkers and histological patterns of CTD-ILD and possible subtypes of CTD-ILD.

### Conclusions

In summary, increased levels of BAFF were found in the circulation of patients with CTD-ILD and UCTD-ILD, in whom serum levels were inversely correlated with lung function. Immunohistochemical studies showed BAFF overexpression in the lung parenchyma in CTD-ILD. Our findings suggest that serum BAFF levels may be clinically useful for distinguishing CTD-ILD from CFIP.

## Abbreviations

CTDs: Connective tissue diseases
CTD-ILD: Connective tissue disease-associated interstitial lung disease
CFIP: Chronic fibrosing interstitial lung disease
FVC: Forced vital capacity
ILDs: Interstitial lung diseases
IPF: Idiopathic pulmonary fibrosis
KL-6: Krebs von den Lungen-6
NSIP: Nonspecific interstitial pneumonia
TNF: Tumour necrosis factor
UIP: Usual interstitial pneumonia
