# Erratum to: Serum B cell–activating factor (BAFF) level in connective tissue disease associated interstitial lung disease

**DOI:** 10.1186/s12890-016-0278-1

**Published:** 2016-08-08

**Authors:** Tsutomu Hamada, Takuya Samukawa, Tomohiro Kumamoto, Kazuhito Hatanaka, Go Tsukuya, Masuki Yamamoto, Kentaro Machida, Masaki Watanabe, Keiko Mizuno, Ikkou Higashimoto, Yoshikazu Inoue, Hiromasa Inoue

**Affiliations:** 1 Department of Pulmonary Medicine, Graduate School of Medical and Dental Sciences, Kagoshima University, 8-35-1 Sakuragaoka, Kagoshima, 890-8520 Japan; 2 Department of Molecular and Cellular Pathology, Graduate School of Medical and Dental Sciences, Kagoshima University, Kagoshima, Japan; 3 Clinical Research Center, National Hospital Organization Kinki-Chuo Chest Medical Center, Osaka, Japan

## Erratum

After the publication of this work [1], it was brought to the authors attention that statements in the article are not consistent with Fig. 1. The statement “Our findings show that patients who met Kinder’s criteria for UCTD had significantly higher serum BAFF levels than patients with CFIP;” and “In summary, increased levels of BAFF were found in the circulation of patients with CTD-ILD and UCTD-ILD, in whom serum levels were inversely correlated with lung function” are not a correct reflection of the results from the data analysis in Fig. 1 of the article.

The corrected statements are provided here as follows: “Our findings show that patients who met Kinder’s criteria for UCTD tended to have higher serum BAFF levels than patients with CFIP;” and “In summary, increased levels of BAFF were found in the circulation of patients with CTD-ILD, in whom serum levels were inversely correlated with lung function.”
